# Suboptimal Vaccination Coverage and Serological Screening in Western Australian Children With Inflammatory Bowel Disease Receiving Immunosuppressive Therapy: An Opportunity for Improvement

**DOI:** 10.7759/cureus.73744

**Published:** 2024-11-15

**Authors:** Muhammad Shahzad Shabir, Sibgha Arif, Dan Yeoh, Zubin Grover

**Affiliations:** 1 Gastroenterology, Perth Children Hospital, Perth, AUS; 2 Family Medicine, Sonic Health Plus, Perth, AUS; 3 Infectious disease, Perth Children Hospital, Perth, AUS

**Keywords:** children, immunization, inflammatory bowel diseases, vaccine-preventable diseases, western australia

## Abstract

Background

Patients with inflammatory bowel disease (IBD) face an increased likelihood of severe illnesses, including those caused by vaccine-preventable diseases. Consequently, the purpose of this study was to evaluate both vaccination rates and serological screening in children with IBD in Western Australia, focusing on compliance with routine and additional vaccines, and pre-treatment screening for infections before starting immunosuppressive (IS) treatment.

Method

The study was conducted at Perth Children’s Hospital (PCH) from June 2021 to February 2022, focusing on children aged 0-18 with confirmed IBD diagnoses. Demographic and medical data were collected and matched with immunization records from the Australian Immunisation Register (AIR) to audit compliance with routine childhood vaccinations and additional vaccines (23-valent pneumococcal, human papillomavirus (HPV), and annual influenza). Data from medical records were analyzed for compliance with serologic testing (QuantiFERON TB, Hep B and C, Varicella, and Epstein-Barr virus (EBV)) before initiating IS therapy, which included immunomodulators, biologics, or small molecules.

Results

Of the 243 patients, 120 (52%) were diagnosed with Crohn’s disease and 106 (43%) with ulcerative colitis. A total of 181 patients (74.5%) were treated with immunomodulators, while 62 (26%) received biologic therapies. Incomplete routine vaccination coverage was identified in 71 (29.2%) patients, with no notable differences observed between the IS and non-IS groups (p=0.3). Specific vaccines with incomplete coverage included HPV in 49 (24%) patients, Varicella in 39 (16%) patients, and diphtheria-tetanus-pertussis (DTP in 16 (6.5%) patients. Pre-treatment serological screening was also suboptimal, with the lowest testing rate for EBV at 32 (13.2) patients and the highest for Varicella at 181 (74.6%) patients.

Conclusion

The results emphasized the importance of targeted interventions to enhance vaccination and screening practices, enhancing disease management, and reducing the possibility of preventable infections in the vulnerable populace.

## Introduction

Individuals with inflammatory bowel disease (IBD) are at a higher risk of developing infections owing to impaired immune system function, affecting both inherent and adaptive immune responses, as well as the use of immunosuppressive (IS) treatments. Nonetheless, many of these infections can be avoided, or their impact mitigated, through vaccination [1,2]. Additional factors contributing to an increased risk of infection include disease activity, malnutrition, and surgical interventions [3,4]. Data from various studies indicate that the prevalence of pediatric IBD has increased on a global scale, with substantial documentation available specifically within Australia [5]. A study conducted in Victoria, Australia, revealed a significant increase in the prevalence of both ulcerative colitis (UC) and Crohn’s disease (CD), with rates rising 11-fold from 1990 to 2010 for UC and 15-fold from 1970 to 2000 for CD [6]. IS treatment is being increasingly applied and initiated at earlier stages in patients with IBD [7].

Current recommendations suggest that children with IBD follow the same established vaccination schedule as their healthy counterparts while avoiding live vaccines during periods of IS therapy [7]. Nonetheless, studies have reported incomplete vaccination coverage among children with IBD [8], ranging from 7% to 78% in countries such as France and Canada [7]. Martinelli and his colleagues demonstrated that children with IBD had poor vaccination status at assessment, which was not subsequently addressed with appropriate immunization recovery efforts. Specifically, vaccination rates at diagnosis were found to be unsatisfactory for measles, mumps, and rubella (89.3%), Haemophilus influenzae (81.9%), meningococcus C (23.5%), chickenpox (18.4%), pneumococcus (18.6%), papillomavirus (5.9%), and rotavirus (1.9%) [9]. Likewise, García-Serrano and his colleagues discovered that vaccination compliance did not surpass 65% for any of the vaccines assessed in their research. They emphasized that raising awareness among both patients and physicians is crucial for improving vaccination rates and minimizing the risk of infections that can be prevented by vaccines [2]. In another study by Crawford et al., the authors observed a 90% compliance rate with routine vaccinations but found low coverage for additional recommended vaccines, including the annual influenza vaccine and pneumococcal booster. In this study, only 5% of patients had received the pneumococcal vaccine, and only 10% had ever received the influenza vaccine [10].

Data on immunization in patients with IBD from Western Australia is limited. Therefore, the objective of this research was to evaluate adherence rates to standard and additional recommended vaccines, as well as serology coverage for Epstein-Barr virus (EBV), Hepatitis B, Hepatitis C, and QuantiFERON TB in children with IBD from Western Australia. This article was presented as a poster at the 6th International Symposium on Paediatric Inflammatory Bowel Disease in Edinburgh on 8th September 2022.

## Materials and methods

Study design and data collection

This retrospective study was performed at Perth Children’s Hospital (PCH), a tertiary care center situated in Western Australia, between June 2021 and February 2022. Patients without a confirmed IBD diagnosis, those with incomplete immunization records, or unrelated co-existing medical conditions that could impact vaccination or serological screening, such as congenital immune deficiencies, were excluded from the study. However, children aged 0 to 18 years with a confirmed diagnosis of IBD and complete immunization and serological screening data available through the Australian Immunisation Register (AIR) and the hospital’s IBD database were included in the analysis. Demographic information, such as age, gender, current and past medications, and treatments, was compared with immunization records from the AIR, which is a national database that systematically tracks all vaccinations administered in Australia. The study assessed routine primary childhood vaccinations along with the administration of additional recommended vaccines. In addition to AIR records, the electronic medical records from the IBD database were reviewed to assess compliance with recommended serologic testing before starting immunosuppression (IS) therapy. Immunosuppression included conventional immunomodulators (IM) like methotrexate, azathioprine, tacrolimus, and biologics or small molecules such as JAK-1 inhibitors. Prior to IS therapy, serologic testing for QuantiFERON TB, Hep B, Hep C, Varicella, and EBV was recommended. Demographic details, including age, sex, disease classification, and treatment exposure, were gathered from a retrospective audit of the hospital’s IBD database. Children with IBD receiving nutritional therapy, a single course of weaning prednisone or budesonide, or aminosalicylates were classified as the non-IS group.

Sample size

A sample size of 243 was determined using a 95% confidence interval and a 6% margin of error.

Ethical consideration

The study received PCH ethics and governance approval, which granted a consent waiver due to its retrospective nature. All protocols were enacted in compliance with the codes established in the Declaration of Helsinki.

Data analysis

The data was analyzed using IBM SPSS Statistics for Windows, Version 27.0. Descriptive statistics (frequencies and percentages) was used to present the data, whereas continuous variable (age) was presented as median (IQR). The Chi-square test was employed to find the association between different parameters. A p-value of ≤ 0.05 was deemed statistically significant.

## Results

The study comprised a total of 243 patients, with 140 (58%) males and 103 (42%) females. Among the diagnoses, 120 (52%) patients were diagnosed with CD, 106 (43%) with UC, and 17 (6%) had unclassified IBD. In terms of treatment, 181 (74.5%) patients were receiving immunomodulators, while 62 (26%) were being treated with biologics. The demographic and clinical attributes are detailed in Table 1.

**Table 1:** Clinical attributes of patients CD: Crohn’s disease; UC: ulcerative colitis; IBD: inflammatory bowel disease; IM: immunomodulators

Variables		Frequency (n)	Percentage (%)
Age (years) (median (IQR))		10 (4.3)	
Age at diagnosis of CD (years) (median (IQR))		11 (4.5)	
Age at diagnosis of UC (years) (median (IQR))		8.3 (4.3)	
Gender	Male	140	58
	Female	103	42
Diagnosis	Crohn’s disease	120	52
	Ulcerative colitis	106	43
	IBD - Unclassified	17	6
Treatment	Immunomodulators	44	25
	Biologics alone	47	26
	Combination of biologics and IM	90	49
Up-to-date vaccination	Yes	187	70.8
	No	54	20.5

Table 2 suggested neither the diagnosis type nor the treatment group has a significant impact on whether patients have complete or incomplete vaccination. This lack of association proposed that factors other than diagnosis or treatment might be influencing vaccination status in this cohort. Further, significant associations were found for meningococcal C, and polio vaccinations, indicating higher completion rates for these vaccines among the studied population.

**Table 2:** Association between diagnosis, treatment and immunization status CD: Crohn’s disease; UC: ulcerative colitis; IBD: inflammatory bowel disease; IS: immunosuppressive therapy; HPV: human papillomavirus; DTP: diphtheria, tetanus, and pertussis; PCV: pneumococcal conjugate vaccine *Chi-square test

Variables		Vaccination status		p-value*
		Complete	Incomplete	
Diagnosis	CD	87	25	0.951
	UC	83	24	
	Unclassified IBD	4	2	
Treatment group	IS	125	56	0.3
	Non-IS	47	15	
Routine vaccination	HPV	185	58	0.341
	Varicella	204	39	0.201
	DTP	227	16	0.791
	PCV	228	15	0.09
	Meningococcal C	229	14	0.03
	Polio	238	5	<0.001

**Figure 1:**
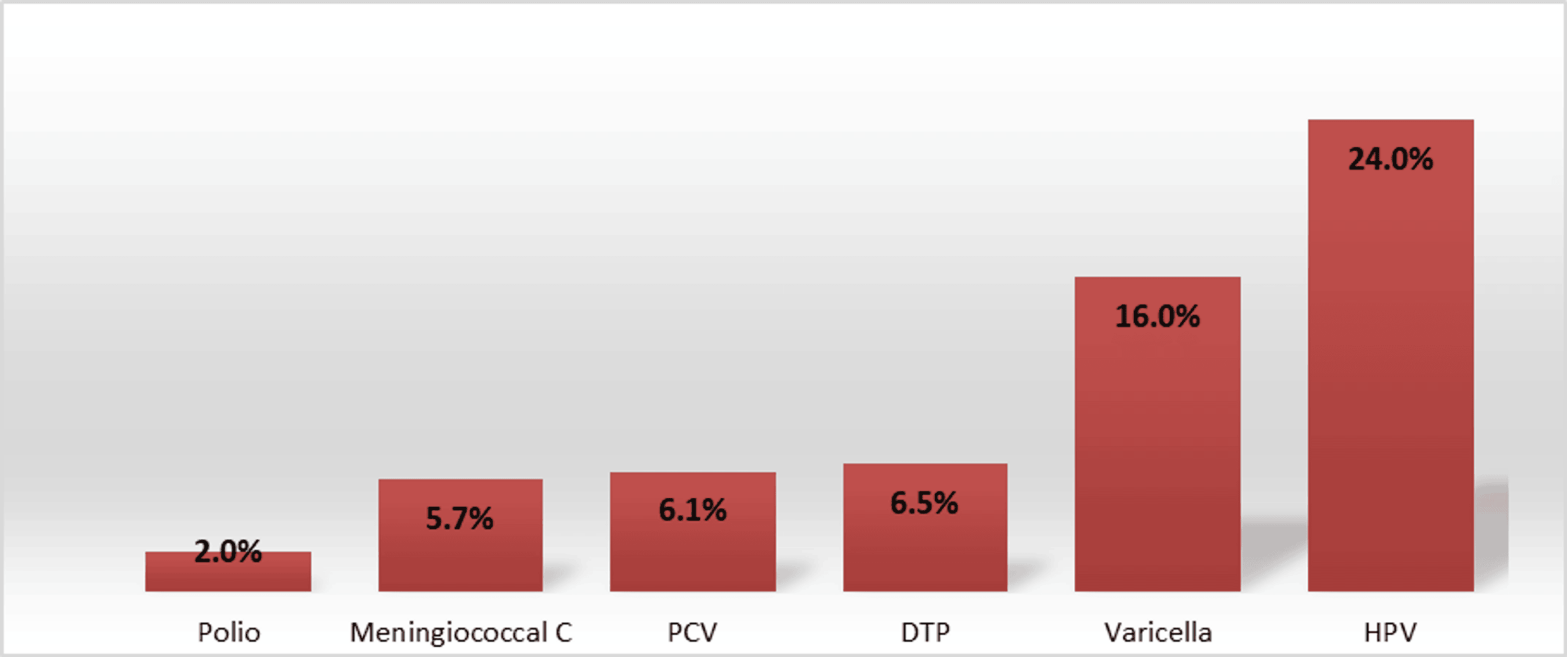
Incomplete coverage rates for routine vaccination among children with IBD in Western Australia PCV: pneumococcal conjugate vaccine; DTP: diphtheria, tetanus, and acellular pertussis; HPV: human papillomavirus; IBD: inflammatory bowel disease

Figure 1 depicts the incomplete vaccination coverage rates for routine immunizations among children with IBD in Western Australia. A total of 71 (29.2%) patients had incomplete coverage for age-appropriate routine vaccinations. No significant difference in incomplete coverage was found between the IS and non-IS groups (56/181 vs. 15/62, p=0.3). Specific vaccines with incomplete coverage included human papillomavirus (HPV) in 49 (24%) patients, Varicella in 39 (16%) patients, and DTP in 16 (6.5%) patients.

**Figure 2:**
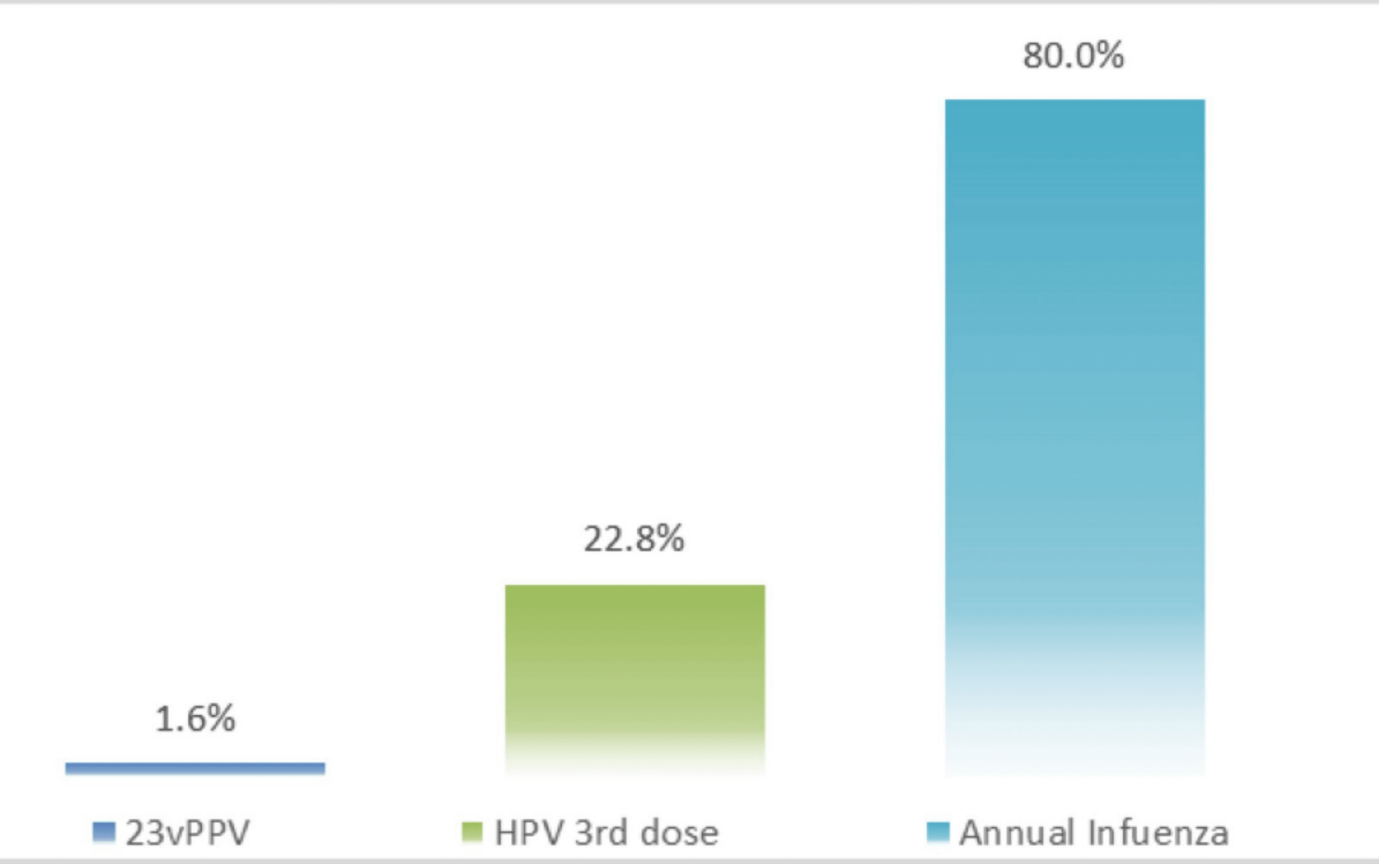
Coverage for additional recommended vaccines for patients on Immunosuppressants PPV: pneumococcal polysaccharide vaccine; HPV: human papillomavirus

Figure 2 highlighted a substantial variance in vaccine coverage, with strong adherence to the annual influenza vaccine (80%) but significantly lower uptake for the 23vPPV (1.6%) and HPV (22.8%) vaccines.

**Figure 3:**
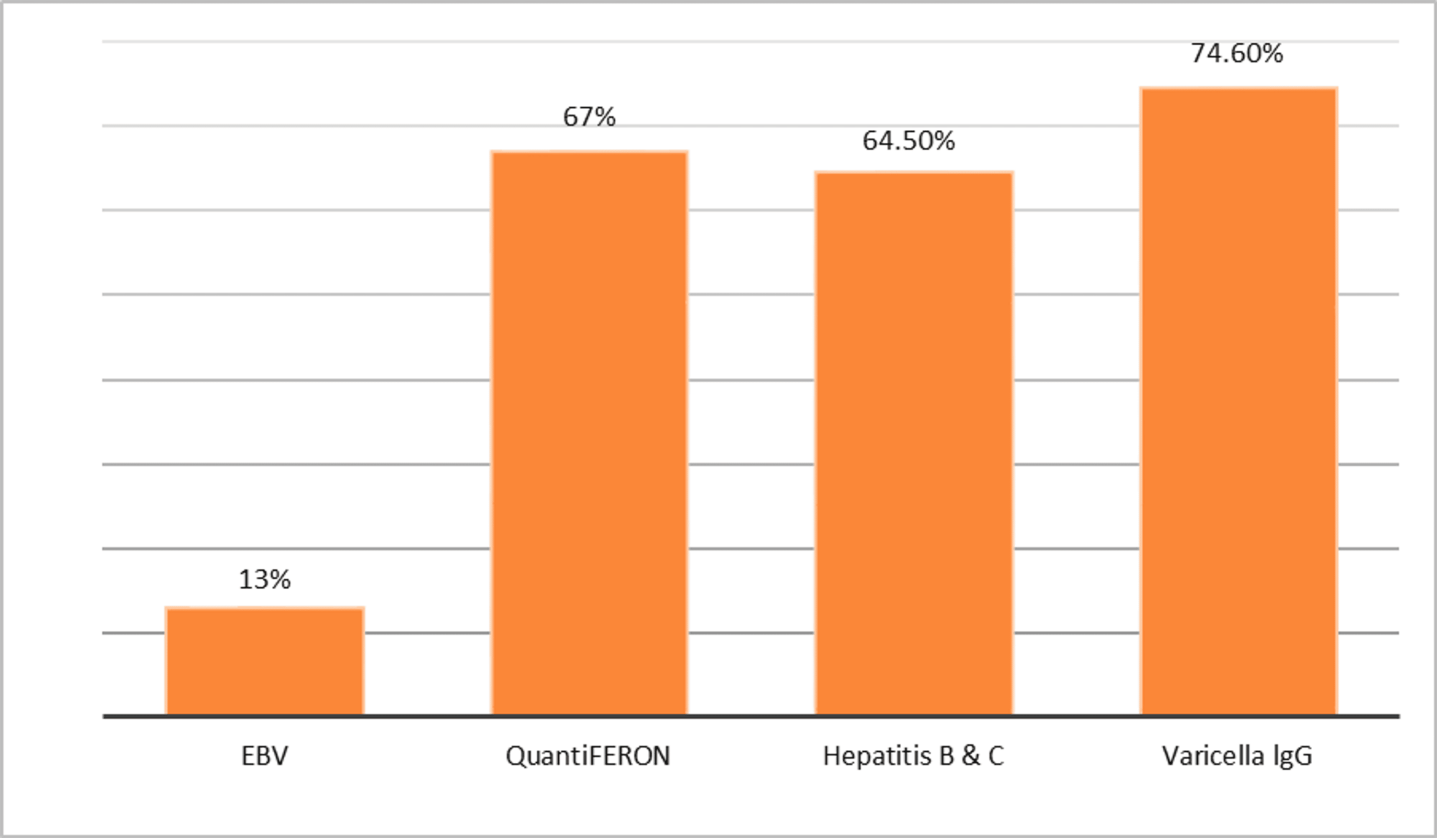
Serological testing before initiating immunomodulators EBV: Epstein-Barr virus

Figure 3 represents the variation in pre-treatment screening for infections among the patients. The lowest testing rate was for EBV, with only 32 (13.2%) patients screened. QuantiFERON testing, used for tuberculosis screening, was conducted in 163 (67%) patients. Screening for Hepatitis B and C was performed in 157 (64.5%) patients. The highest testing rate was for Varicella IgG, with 181 (74.6) patients undergoing this test prior to starting immunomodulators.

## Discussion

Individuals with IBD are at risk of developing opportunistic infections [11]. However, the occurrence of IBDs, which include CD and UC, is rising in the pediatric population, with current rates estimated at 10 cases per 100,000 children [12] and 10-20% of all new IBD cases globally [13]. Regardless of recommendations in clinical guidelines for vaccinating patients with IBD, awareness about vaccinations remains inadequate. The low vaccination rates among IBD patients can be linked to physicians’ limited awareness, uncertainties about the effectiveness of vaccines, and concerns over possible side effects [14].

This study identified CD in 120 (52%) patients, which occurred at a higher rate compared to UC, observed in 106 (43%) patients, aligning with an Australian study that also reported higher CD rates than UC, with respective frequencies of 165.5 and 131.4 per 100,000 [15]. However, contrary findings were presented by Busingye et al., who documented slightly higher rates of UC (334 per 100,000) compared to CD (306 per 100,000) [16]. Similarly, Hein et al. reported a higher prevalence of UC (412 per 100,000) than CD (322 per 100,000), demonstrating variation in the global prevalence of these inflammatory bowel diseases [17].

In the present study, only 170 (70%) children in the IBD cohort had received all the routine vaccines. This finding was supported by Longuet et al. wherein 66% of children with IBD were fully vaccinated according to the national recommendations [18]. Contrarily, Soon et al. found that about 89.7% of children with IBD received all routine childhood immunizations [19]. Kowalska-Duplaga and her research team also assessed 267 children in their study, which included 214 children diagnosed with IBD and 53 healthy controls. The study indicated that none of the participants had completely fulfilled the standard childhood vaccination program recommended in Poland [20].

The current finding demonstrated that additional immunizations like HPV, annual influenza vaccination, and pneumococcal vaccine boosters were often missed in children with IBD. This aligns with the observations of Holland et al. who found that a significant number of individuals with IBD were not receiving key vaccinations, particularly the seasonal influenza vaccine, as well as missing doses from the HPV series and the pneumococcal polysaccharide vaccine (PPSV23), which is specifically recommended for those who are immunosuppressed. Among the 45 IBD patients studied, 24% had received the first dose of the HPV vaccine but had not completed the second dose in the series [12]. Similarly, Ford and his co-workers found that only 43.5% of patients had successfully completed the vaccination schedule recommended by the 12-month mark [5]. Another study by Banaszkiewicz and his colleagues indicated that influenza immunization rates were lower (7.8%) in pediatric IBD patients compared to the general pediatric population (18.3%), with fear of adverse reactions to the vaccine recognized as the most common reason [21]. Fear of disease activation is also often cited as a reason for poor immunization coverage, which can be allayed by proper counseling [18].

This study demonstrated serologic screening rates ranging from 157 to 181 patients, accounting for 64.5% to 74.6% of the cohort. There is often a lack of focus on immunization by pediatric gastroenterologists who manage children with IBD, as they tend to believe this responsibility falls under the care of the primary pediatrician or general practitioner. A large survey of 657 pediatric gastroenterologists from North America found that only 63.5% assessed patients’ immunization status at diagnosis, and a mere 9% routinely ordered serological tests to evaluate immunity against vaccine-preventable diseases. This underscores a need for improved coordination between specialists and primary care providers to ensure comprehensive vaccination and screening [22].

One approach to enhancing vaccination rates is by referring all newly diagnosed patients to a specialist immunization service. For instance, a study conducted at the Royal Children’s Hospital in Melbourne demonstrated that within 12 months of referral, 92.8% of patients received additional vaccinations [5]. Similarly, educational programs that emphasize the importance of vaccines could also improve compliance. In a Canadian study, after providing education and counseling, the rate of annual influenza vaccination increased from 47% to 89.5%. These examples highlight how targeted interventions and education can significantly boost vaccination adherence in patients with chronic conditions [23].

Limitations

This study has certain limitations, primarily due to its exclusive reliance on the AIR to assess vaccination status. While the AIR may not capture every instance of vaccination, it provides a reasonable estimate. Additionally, for serological screening, only our hospital’s electronic pathology database was reviewed, meaning that some serology tests conducted outside our pathology system could have been missed. Furthermore, the study did not investigate the barriers contributing to non-compliance with additional vaccinations or pre-IS therapy serological screening. It also did not identify any potential contraindications to routine or additional vaccines. Thus, future research must improve collaboration among healthcare professionals to tackle these issues. Opportunities for improvement include enhanced patient education, ongoing training for healthcare professionals, and the establishment of specialized vaccination clinics to boost compliance with both serologic screening and vaccination among children with IBD.

## Conclusions

This study demonstrated reasonable coverage for routine vaccinations but highlighted poor adherence to additional recommended vaccines among children on IS, biologics, or a combination of both. The findings emphasized the need for a structured and formal review of vaccination status at the time of IBD diagnosis, as well as before and during the initiation of immunomodulator or biologic therapy. This would allow for timely catch-up vaccinations and serological testing. Incorporating vaccine checklists and serological screening into the IBD care pathway could significantly enhance compliance rates and ensure better protection against preventable diseases.
